# Quantitative Biology of Human Shelterin and Telomerase: Searching for the Weakest Point

**DOI:** 10.3390/ijms20133186

**Published:** 2019-06-28

**Authors:** Pavel Veverka, Tomáš Janovič, Ctirad Hofr

**Affiliations:** LifeB, Chromatin Molecular Complexes, CEITEC and Functional Genomics and Proteomics, National Centre for Biomolecular Research, Faculty of Science, Masaryk University, CZ-62500 Brno, Czech Republic

**Keywords:** telomerase, shelterin, telomere, quantitative biology, protein-protein interaction, protein-DNA interaction, assembly, inhibitor, anticancer

## Abstract

The repetitive telomeric DNA at chromosome ends is protected from unwanted repair by telomere-associated proteins, which form the shelterin complex in mammals. Recent works have provided new insights into the mechanisms of how human shelterin assembles and recruits telomerase to telomeres. Inhibition of telomerase activity and telomerase recruitment to chromosome ends is a promising target for anticancer therapy. Here, we summarize results of quantitative assessments and newly emerged structural information along with the status of the most promising approaches to telomerase inhibition in cancer cells. We focus on the mechanism of shelterin assembly and the mechanisms of how shelterin affects telomerase recruitment to telomeres, addressing the conceptual dilemma of how shelterin allows telomerase action and regulates other essential processes. We evaluate how the identified critical interactions of telomerase and shelterin might be elucidated in future research of new anticancer strategies.

## 1. Introduction

Telomeres are an excellent example of how a combination of random mutations and selection over hundreds of millions of years solves challenges that appear during evolution.

The first challenge is that the ends of linear DNA of eukaryotic chromosomes cannot be fully replicated. The incomplete replication of the 3′ end of DNA results in an overhang of single-stranded telomeric DNA. The lagging strand, and thus the length of telomeric DNA, shortens every round of replication (50–200 per each DNA synthesis). Even though the gradual shortening of telomeric DNA could be used as a molecular mark of the number of divisions that a cell has undergone, the gradual loss of DNA sequence should be prevented in fast-dividing germ line cells and stem cells [1]. The end-replication problem is solved by the action of telomerase. Telomerase is a nucleoprotein complex, containing an RNA template, that synthesizes tandem repeats of telomeric DNA and compensates for the DNA erosion.

Another challenge is that chromosomes have to be protected against end-to-end fusions and undesirable recognition of telomeric DNA ends by double-strand DNA break repair pathways. To achieve chromosome protection, a protein complex called shelterin binds telomeric DNA. Shelterin not only protects the DNA ends, but also regulates the access of processing enzymes, such as telomerase and helicases, to telomeric DNA.

Finally, the fact that telomerase is also active in tumor cells, contributing to their immortalization, could be turned into an advantage in the treatment of cancer if we could deactivate or slow down telomerase, specifically in tumor cells, we would be able to eradicate tumor cells more effectively.

In this review, we focused on new findings on how shelterin assembles and mediates telomerase recruitment to telomeres and how the interaction map of shelterin and telomerase could be employed in anticancer strategies of new biomolecular therapies. 

## 2. Shelterin Structure and Binding Features

Human chromosome ends are bound to, and protected by, shelterin complexes. Shelterin consists of six different proteins: telomeric repeat-binding factor 1 (TRF1; also known as TERF1), telomeric repeat-binding factor 2 (TRF2; also known as TERF2), repressor and activator protein 1 (RAP1; also known as TERF2IP), TRF1-interacting nuclear protein 2 (TIN2; also known as TINF2), protection of telomeres 1 (POT1), and TPP1 (derived from its former names TINT1, PTOP, PIP1; also known as ACD).

**Figure 1:**
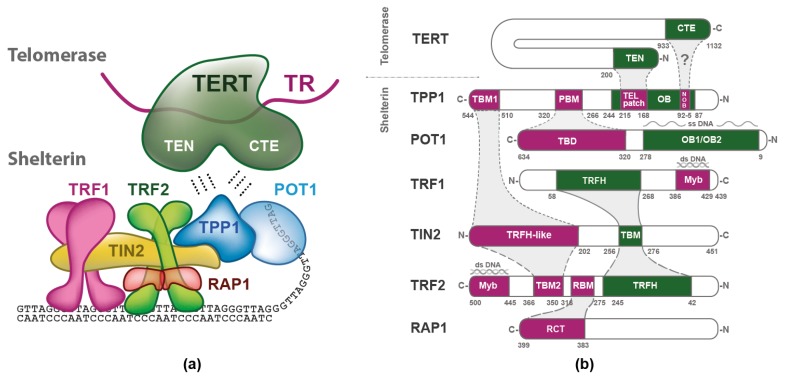
Arrangement and interaction domain map of shelterin and telomerase: (**a**) Schematic description of telomerase and shelterin subunits. TERT is the catalytic subunit with telomerase essential N-terminal (TEN) domain and C-terminal extension (CTE) domain, TR is the RNA subunit of telomerase. Shelterin subunits TRF1, TRF2, and RAP1 are shown as dimers, and the other shelterin subunits are illustrated as monomers. (**b**) The interaction map of the telomerase TERT subunit and the six human shelterin subunits are depicted. The Myb/SANT domains of TRF1 and TRF2 recognizing double-stranded DNA are indicated with Myb. The OB1/OB2 domain of POT1 recognizing single-stranded DNA. TBD is the TPP1 binding domain of POT1. PBM is POT1 binding motif of TPP1. The OB domain of TPP1 contains two regions: the TEL patch that binds TEN domain of telomerase and the NOB region that probably binds CTE domain of telomerase, denoted by “?” [9]. TBM of TIN2 binds the TRFH domain of TRF1 and TRF2. TBM1—TIN2-binding motif of TPP1, TRFH—dimerization domain of TRF1 and TRF2, TRFH-like—TIN2 domain that binds TPP1 and TRF2; TBM2 binds TRFH-like binding motif of TIN2, and TBM2—TIN2-binding motif of TRF2. RBM—RAP1 binding motif of TRF2. RCT is the RAP1 C-terminal domain that binds RBM of TRF2. The more solid the outline between the interacting domains, the higher their mutual affinity [7,10].

Importantly, a stable shelterin complex is formed solely through protein-protein interactions of the shelterin subunits, and DNA is not required [2,3]. As shelterin functions on telomeric DNA that contains a single-stranded overhang, the shelterin anatomy can be divided into two parts that associate with either double- or single-stranded telomeric DNA (Figure 1a). The part of shelterin that binds the double-stranded telomeric DNA consists of TRF1, TRF2, RAP1, and TIN2 [4]. Both TRF1 and TRF2 bind telomeric DNA duplexes specifically through the Myb domain [5]. The next subunit, RAP1 interacts solely with TRF2. RAP1 binds TRF2 tightly with an affinity similar to the affinity of TRF2 to DNA [6]. RAP1 also prevents the positively charged basic domain of TRF2 from sequence-nonspecific DNA binding, thereby increasing the specificity of TRF2 for human telomeric TTAGGG repeats [6]. TIN2 interconnects TRF1 and TRF2, and enhances TRF2 stability at telomeres [7,8].

The part of shelterin that is associated with single-stranded DNA consists of the heterodimer POT1 and TPP1. POT1 binds single-stranded telomeric DNA via two oligonucleotide/oligosaccharide binding (OB) folds (Figure 1b) [11,12]. POT1 recognizes the sequence 5′-TTAGGGTTAG and binds it with a nanomolar affinity when located at the 3′ end, or with a slightly lower affinity when the recognition sequence is at an internal position [11,13].

Recently, the Cech laboratory carried out biophysical studies describing how the single-stranded and double-stranded DNA binding parts of shelterin contribute to the DNA-binding affinity of shelterin [14]. Lim et al. designed a DNA substrate, comprising both single-stranded and double-stranded telomeric regions separated by 8 bp of random double-stranded DNA. When the double-stranded telomeric sequence on DNA was replaced with a random DNA sequence, the original nanomolar binding affinity of shelterin core proteins TRF2-TIN2-TPP1-POT1 dropped 6-fold. Similarly, when the part of the single-stranded DNA with telomeric sequence was replaced with a random DNA sequence, the binding affinity of the core shelterin dropped 35-fold. Interestingly, after removal of either TRF2 or POT1 from the core shelterin, its binding affinity to the DNA substrate decreased 50-fold and 14-fold, respectively [14]. These results suggest that the removal of TRF2 or POT1 from shelterin decreased its DNA binding affinity. The quantitative experiments also showed the contribution of TRF2 and POT1 to the specific recognition of telomeric DNA by shelterin. In addition, this is in accordance with the general view that DNA-binding proteins associate with DNA nonspecifically at first and seek their recognition sequence and optimal binding site through specific interactions subsequently [15].

**Figure 2:**
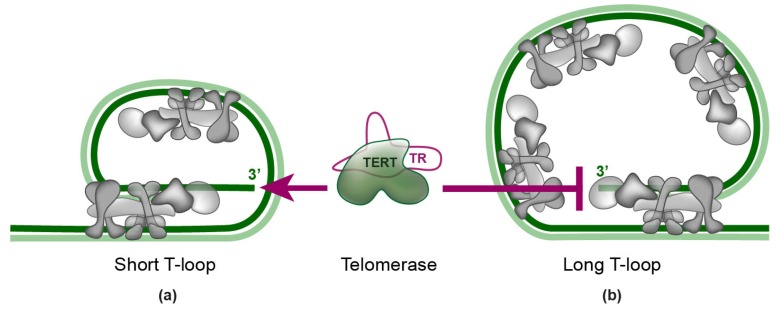
T-loop formation as a factor for telomere length regulation. The 3′ overhang invades the double-stranded telomeric DNA and, by base-pairing with the C-strand, displaces the G-strand, which causes the end of the telomere to be tucked in. Double-stranded DNA-binding proteins of shelterin, TRF1 and TRF2, mediate and fold DNA to the t-loop. The amount of shelterin that is bound to telomeres is dependent on the t-loop length. (**a**) A short t-loop with less abundant shelterin has a lower probability that shelterin covers and hides the 3′ overhang of telomeric DNA. Telomerase can extend telomeric DNA. (**b**) The long t-loop is occupied by more shelterin complexes, so the probability that the 3′ overhang is covered and hidden is higher. Telomerase access to the 3′ end single-stranded DNA is blocked and no DNA extension occurs.

Shelterin complexes appear to bind telomeric DNA without recruiting each other in vitro [2], which is in accordance with the observation that shelterin finds its binding sites through a diffusive 3D search, as well as by a 2D search along the DNA [2,4]. Telomeric single-stranded overhang can fold back and form a telomeric loop (t-loop). This structure was first observed by electron microscopy [16] and later confirmed by super-resolution microscopy [17]. T-loops can physically hide the very ends of eukaryotic chromosomes from DNA repair pathways and telomerase [18]. TRF2 is essential for the formation of the t-loop [17]. In addition, POT1 stabilizes t-loop formation [19]. T-loops are protected and maintained by shelterin complexes [20]. The putative arrangement of shelterin on t-loops is shown in Figure 2. Recently, F. Erdel et al. have investigated the localization of shelterin on telomeric DNA in vitro. They discovered that shelterin rapidly and primarily recognizes telomeric repeats by a three-dimensional diffusive search. The complex remained bound to telomeric DNA more than 30-fold longer in comparison with non-telomeric DNA—it persisted bound for ten minutes without dissociating from telomeric DNA. According to their electron microscopy experiments, there is approximately one shelterin complex per 100 bp of telomeric repeats [2].

## 3. Telomerase

Telomerase consists of two main subunits. One of the subunits is the telomerase reverse transcriptase (TERT). TERT is recruited to telomeres during the S-phase of the cell cycle. The interaction of telomerase with shelterin is mediated by TPP1 (Figure 1). The other subunit of telomerase is telomerase RNA (TR)—a 451 nucleotides-long oligomer—which binds the TERT subunit by the template-pseudoknot domain and CR4-CR5 domain [21,22,23,24]. TR has also been shown to contribute to TERT processivity, as TR also provides a template boundary element that limits the extent of reverse transcription [25,26]. The t-loop model of telomere and shelterin arrangements suggests that telomerase access to single-stranded telomeric 3′ overhang contributes to the regulation of the telomere length maintenance (Figure 2) [20].

Telomerase is closely associated with cancer and other diseases. Telomerase is extremely important for tumorigenesis—telomerase is activated in 90% of all malignant tumors [27]. In many cancer cells, mutations in the promoter of the gene that encodes for telomerase reverse transcriptase (TERT) causes higher expression rate of TERT [28,29]. In addition, mutations with loss-of-function in either TERT or TR have been associated with dyskeratosis congenita, aplastic anemia and pulmonary fibrosis [30].

### 3.1. Structure of TERT and TR

The TERT catalytic core (1132 amino acids) consists of four major domains: the telomerase essential N-terminal (TEN) domain, the high-affinity RNA-binding domain (TRBD), the reverse transcriptase (RT) domain, and a C-terminal extension (CTE), analogous to a polymerase thumb [31,32,33]. The 3′ ends of TR contain an H/ACA Cajal box (CAB) motif that can bind various telomerase accessory proteins. TR is associated with RNA-binding proteins dyskerin, NHP2, NOP10, and GAR1, which ensure TR stability, 3D structure of RNA, and telomerase ribonucleoprotein biogenesis [34,35].

Recently, Nguyen et al. showed in detail the cryo-EM structure of the active TERT protein subunits and TR, illuminating the structural basis of TR motif functions and human disease mutations that cause telomerase deficiency. They used transient transfection of HEK 293T cells with vectors expressing TERT and TR to prepare enough telomerase complexes for cryo-EM. To determine the telomerase structure at subnanometer resolution, they fit EM densitograms with available high-resolution structures and homology models of telomerase complex components [36]. The structure data supports the hypothesis that telomerase from various organisms functions in a monomeric state [37,38]. The monomeric state of telomerase, consisting of one TERT and one TR, is in agreement with suggestions of an evolutionarily-conserved single-TERT catalytic core [36,37,39,40].

The TEN domain of TERT has been implicated in providing the ‘anchor site’ that binds telomeric DNA upstream from the primer-template interaction [41,42,43]. The TEN domain also contains the DAT motif, which has been postulated to be involved in telomerase recruitment to the shelterin complex [44]. In particular, the mutation K78E in the DAT domain was shown to prevent telomerase recruitment by TPP1 [45].

There are two TR domains critical for telomerase activity: the first is the domain with the template and adjacent pseudoknot (t/PK), and the second is the conserved regions 4 and 5 (CR4/5), consisting of a branched junction of stems P5 and P6 with the activity-critical stem-loop P6.1 [46,47]. The pseudoknot and triple helix structure contribute to catalysis, perhaps by orienting the primer-template duplex in the enzyme active site [48,49,50].

### 3.2. The Function of TERT and TR

In vivo, the telomerase action on DNA can be described by: (i) binding frequency—the number of bindings to telomeric DNA per cell cycle and (ii) repeat addition processivity—the number of newly synthetized telomeric DNA repeats per association event [45].

Telomerase interacts with the chromosome end in two different ways, namely: (i) short “probing” interactions—dynamic interactions with telomeres and (ii) “static” interactions—sufficiently long interactions that allow telomere elongation. TERT requires TPP1 binding for both interaction modes [45,51,52]. Recently, experiments with live cell imaging demonstrated that long-static interactions of telomerase occur only when telomerase pairs with a single-stranded DNA overhang [45].

In vivo, telomerase can add around 50–60 nucleotides to most chromosome ends in a single processive step [53]. In vitro, telomerase has a higher processivity and can recover 100 nucleotides lost from replicated chromosome ends in a single elongation event [33]. If TERT is substantially overexpressed, the TR subunit becomes limiting, so telomerase activity increases only ∼1.5–2-fold [45,52,54,55]. 

The association of TCAB1 with TR is required for telomerase localization in Cajal bodies [56]. Cajal bodies are regions within the nucleus that are rich in proteins and RNAs that are involved in the assembly of ribonucleoproteins. Schmidt et al. revealed how telomerase explores the nuclear space to search for telomeres through dynamic probing interactions using live cell imaging. More than 85% of unbound telomerase moved fast (diffusion coefficient D > 0.3 µm^2^/s) [51]. After association with telomeres, TERT slowed down and appeared in the highly static state with a diffusion coefficient more than 100-fold lower. Similarly, when TERT, which contains TR, is near Cajal bodies, TERT appeared in the static state again [51]. Thus, recent live cell imaging studies suggest that telomerase recruitment to telomeres is driven by dynamic interactions between the rapidly diffusing telomerase and the chromosome ends [51].

## 4. Telomerase Recruitment via Shelterin

Telomerase recruitment to telomeres involves the deployment of telomerase from Cajal bodies to telomeres, which is driven by the interaction of telomerase with TPP1 [57].

The initial in vivo studies suggested that telomerase interacts through TEN and the CTE domain with the OB domain of TPP1 [58]. Specific loop residues within the OB-fold that are critical for the association of TPP1 and telomerase have been identified by structural studies and confirmed by mutation analysis of the OB domain of TPP1 with subsequent quantitative analysis of telomerase activity [59]. The experiment identified seven residues of the OB domain of TPP1 that are critical for telomerase recruitment. The critical residues of TPP1 OB domain define the TEL (TPP1 glutamate (E) and leucine (L)-rich) patch. The four glutamic acid residues give the TEL patch a negative charge. Recently, the Nandakumar laboratory suggested that the highly basic TEN domain of telomerase binds into the acidic pocket of the TEL patch, while the hydrophobic pocket of the N-terminus of the OB domain is occupied by residues residing elsewhere in TERT, probably in CTE [9]. Telomerase recruitment also depends on TIN2, as TIN2 is essential for TPP1 recruitment to telomeres [60].

Recently, the Cech laboratory demonstrated that the full-length POT1, together with the full-length TPP1 protein and the TIN2 subunit, increases telomerase processivity about 3-fold. Lim et al., in their recent study, proposed that the recruitment of telomerase to telomeric DNA could occur not only through the TPP1-POT1 heterodimer, but also through the TPP1-TIN2-TRF1 or the TPP1-TIN2-TRF2 complexes that are associated with the double-stranded DNA-binding part of shelterin [14].

Shelterin proteins TRF1 and TRF2 might play an important role in the protein-counting model of telomere length regulation via telomerase. This model involves two different states: (i) “open” state—telomerase can elongate the telomeres and (ii) “closed” state—telomerase access is blocked (longer telomeres can recruit more TRF1 and TRF2) [61]. Switching between these two states is driven by TRF1 and TRF2 that act as negative regulators of telomere length [61]. Originally, the negative feedback loop for telomere length regulation was suggested in yeast by the Shore laboratory [62]. The protein-counting model is also applicable for t-loop formation and telomere protection by bound shelterin complexes (Figure 2).

## 5. Quantitative Description of Interactions within Shelterin

Shelterin, as a multi-subunit protein complex, is formed through dynamic interactions between its protein subunits. It is known that both TRF1 and TRF2 proteins interact with TIN2, which is the key bridging factor that brings together shelterin subunits attached to double-stranded DNA and subunits attached to the single-stranded DNA overhang.

TPP1 interacts with TIN2, and this particular interaction is suggested to be the essential step in high-order shelterin assembly. The TRF2-TIN2-TPP1 interaction was thoroughly studied by Hu et al. in 2017, where they revealed the importance of TIN2 interactions with TPP1 together with TRF2. They found that the TBM1 (TIN2-binding motif) of TPP1 and TRF2 (TBM2) bind to different sites in TIN2 (2–202aa), where a long alpha helix of TIN2 interconnects TPP1 and TRF2. Thus, it was proposed that the alpha helix acts like a seesaw where TPP1 is on one end and TRF2 is on the other. This concurrent interaction allosterically regulates optimal TIN2 ability to bind TPP1 and TRF2 simultaneously [10]. Recently, we found that, at the single-molecule level, TRF1 is able to replace TRF2 from TIN2 when TPP1 is absent. Thus, TPP1 is required for stable TRF1-TIN2-TRF2 complex formation [3].

Structural studies of shelterin subunits have to be accompanied by affinity studies to reveal structure-function relationships among shelterin subunits. In addition, the description of mutual affinities of shelterin subunits can determine limiting steps in shelterin assembly. Most of the available data are results of binary interaction studies of shelterin components, often limited to their interaction domains only [20,63,64]. When Lim et al. thoroughly investigated the reconstitution of human core shelterin complexes, they revealed a 2:1 binding stoichiometry for TRF2 and TIN2, respectively [14]. 

Rice et al. described the interaction of TPP1 (255–337aa) and POT1 (330–634aa) by isothermal titration calorimetry and revealed that Kd = 120 nM [65]. Previously, it has been shown by the Cech laboratory that TPP1 promotes POT1 binding to single-stranded telomeric DNA [66,67,68]. Accordingly, the Skordalakes laboratory used fluorescence polarization to demonstrate that full-length POT1 binds DNA with a Kd of approximately 20 nM, but, when POT1 is complexed with TPP1 (87–544aa), the affinity increases more than 3-fold [65].

TIN2 has two binding sites with different binding preferences for TRF2. According to Hu et al., 2017, the domain interaction studies using fluorescence polarization revealed that Kd = 2.7 µM for TRF2’s TBM2 domain binding to TIN2 (2–202aa)—truncated protein variants, which covers the TIN2 interaction region for binding TRF1 and TRF2. When TIN2 (2–202aa) is complexed with TPP1′s TBM1 domain before the interaction with TRF2′s TBM2 domain, the affinity is increased 2.5-fold (Kd = 1.1 µM). If we look at the binding of interacting domains the other way around, TIN2 (2–202aa) binds TPP1’s TBM1 domain with Kd = 0.52 µM, and Kd = 0.14 µM when TPP1′s TBM1 domain is complexed with TRF2′s TBM2 domain [10]. TPP1 changes the structure of TIN2 and expands its binding capacity [3,10].

**Figure 3:**
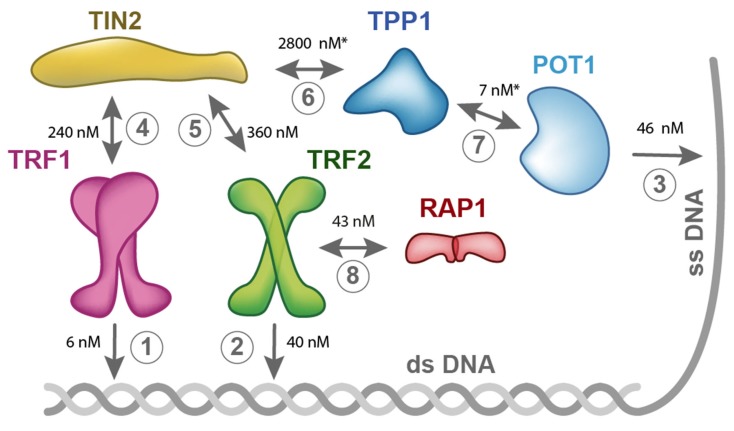
Binary interactions within shelterin measured in our laboratory. Binding studies with six shelterin proteins were carried out using fluorescence anisotropy (1, 2, 3, 7, 8) and thermophoresis (4, 5, 6). The asterisks denote that the Kd values were determined for truncated variant of TPP1 (89–544aa) and POT1 (312–634aa). All other quantifications of interactions were carried out with full-length proteins. The binding buffer was 50 mM sodium phosphate, pH 7.0, 50 mM NaCl. The binding temperature was 25 °C, except for 1 where 10 °C was used to secure TRF1 protein stability during measurements. Kd values were determined as the mean of at least three independent experiments with a relative standard deviation of less than 10%.

We have been studying mutual interactions of human telomeric proteins and DNA in our laboratory systematically [3,6,68,69]. Figure 3 summarizes the results of our binary interaction studies with proteins of human shelterin. Our recent thermophoresis measurements with full-length proteins have revealed that TRF1 binds to TIN2 with Kd = 240 nM, whereas TRF2 binds to TIN2 with Kd = 360 nM. (Figure 3). These data suggest that TRF1 shows greater binding affinity to TIN2 than TRF2. Based on our quantification of mutual interactions within shelterin, we propose that the limiting step for the complete shelterin complex assembly is the interaction of TIN2 and TPP1. The binding of full-length TIN2 and TPP1 (89–544aa) is characterized with the highest Kd value (2.8 µM), i.e., the lowest affinity, measured by fluorescence anisotropy and thermophoresis. Thus, the interaction of TIN2 and TPP1 is the weakest interconnection within the shelterin complex. The interaction of TIN2 and TPP1 plays a crucial role in bridging shelterin parts attached to single and double-stranded telomeric DNA (Figure 1a, Figure 2, and Figure 3). It is important to point out that mutual affinity of two shelterin proteins might be potentiated by binding of other shelterin components. 

The quantitative description of shelterin subunit interactions suggests that the weakest point is protein TIN2 and its TIN2-TPP1 interaction. In addition, the other weak point is the binding of telomerase by TPP1, as telomerase recruitment is a crucial step in the telomere length maintenance mechanism. Thus, TPP1 participates in the interactions that are limiting for shelterin assembly, on one hand, and telomerase recruitment, on the other hand.

## 6. Implication for Anticancer Drug Design

The inhibition of telomerase activity may increase the sensitivity of tumor cells to chemotherapy, radiation, and other targeted therapies. In order to slow down telomere elongation, we could block the interaction between telomerase and shelterin. To impair the telomerase function completely, telomerase binding to DNA would have to be abolished. Two promising approaches are being studied: (i) block the TR-binding region of telomerase (e.g., with inhibitor GRN163L) or (ii) overprotect single-stranded telomeric DNA overhang by its hybridization with artificial T-oligonucleotides. Both inhibitory strategies have demonstrated promising anticancer activity in multiple cancer types via the induction of potent DNA damage responses [70,71]. 

However, the main cause of cancer cells’ eradication is the shortening of telomeres, and not telomerase inhibition exclusively, as cell may initiate telomerase-independent ways of telomere length maintenance. When telomere length reaches a critically short length, cells enter replicative senescence [72]. The deleterious effect of telomere shortening in cancer cells can be triggered by treating cells with cytostatic drugs. 

### 6.1. GRN163L (Imetelstat)

GRN163L (JNJ-63935937, also known as Imetelstat) has been abundantly studied as a competitive inhibitor of telomeric DNA binding [45].

GRN163L has a unique telomerase inhibition ability: it binds directly and tightly to telomerase TR template. The telomerase RNA subunit represents an attractive target for oligonucleotide-based inhibition. The RNA sequence of TR contains an 11-nucleotide-long template region for binding and extending telomeric DNA substrates that is easily accessible for hybridization with complementary oligonucleotides [73].

GRN163L contains a complementary sequence to human TR (5′-palmitate-TAGGGTTAGACAA-NH_2_-3′). Thus, GRN163L serves as a direct antagonist to the telomerase RNA template TR [71,74,75].

GRN163L has high solubility in aqueous solutions, it is resistant to nucleases, shows high stability in acidic solutions or in the presence of metabolites, and demonstrates high thermal stability of duplexes formed with the complementary RNA strands [76,77,78,79,80]. Importantly, GRN163L has been shown to significantly suppress not only the growth of primary tumors, but also the spread and proliferation of metastases [71]. GRN163L progressively diminishes the amount of telomeric primer bound to telomerase, consistent with the hypothesis that GRN163L competes with single-stranded telomeric DNA for telomerase binding [45]. The inhibition of telomerase activity in cancer cells by GRN163L results in progressive shortening of telomeres and eventual senescence or apoptosis [80].

### 6.2. Clinical Studies of GRN163L

To date, GRN163L is in the second phase of clinical trials, according to the Drugbank database. Systematic studies of GRN163L may reveal other therapeutic effects beyond the inhibition of metastasis and cancer proliferation, for example, involving the immune system and reduction of angiogenesis. Reversible effects were revealed when stem cells were shortly treated with GRN163L in vitro [81]. Despite numerous experimental studies showing promising anticancer effects of GRN163L, FDA approval has not been received yet. It is probably because clinically relevant doses of GRN163L as a stand-alone therapy showed an elevated toxicity level [82,83]. Some patients with a pediatric brain tumor suffered from hematological toxicity of GRN163L—the most common side effects were thrombocytopenia, lymphopenia, and neutropenia [82]. GRN163L may have several side effects on the stem cell population [81]. On the other hand, GRN163L showed promising results in combination with radiation therapy, which may overall improve the therapeutic index [84,85,86]. Breast cancer cells treated with GRN163L showed enhanced sensitivity to ionizing radiation that reduced their survival by an additional 30% [84].

### 6.3. T-oligo Cancer Therapy

T-oligo (an oligonucleotide homologous to the 3′-telomere overhang) targets abnormal regulatory signaling pathways, including DNA damage responses (DDRs) in cancer cells [87,88,89,90,91]. It has no or minimal toxicity in mice, melanocytes, or other normal cells [74,91,92,93,94]. After exogenous addition and accumulation in the nucleus, T-oligos induce DDRs, such as apoptosis and cell cycle arrest, which can be facilitated by the ataxia telangiectasia mutated pathway and its effectors cdk2, E2F1, p53, p95/NBS1, and pRb in cancer cells [74,91,93,94,95]. T-oligo has anticancer activity in multiple cancer cell lines in vitro, such as breast, colorectal, lung, lymphoma, melanoma, ovarian, pancreatic, and prostate cancers [86,87,88,89,90,91,95,96].

Ivancich et al. suggested two main models of the mechanism of anticancer effects by T-oligos [97]. The first model—“exposed telomere mimicry model”—hypothesizes that DNA damage-sensing proteins detect the T-oligo that accumulates in the nucleus of a cancer cell due to its homology to the telomere. The second model—“shelterin dissociation model”—is where T-oligo interacts with shelterin proteins, which are dissociated from the telomere and thus T-oligo exposes the telomere overhang that can initiate DNA damage responses [97]. Despite the initial promising results, T-oligos have not proceeded to clinical trials yet [71].

## 7. Perspectives

So far, the quantitative descriptions of binding affinities among shelterin subunits suggest that the weakest point of shelterin assembly is the TIN2-TPP1 interaction. The other potential weak point is the TPP-mediated recruitment of telomerase. Hence, TPP1 is engaged in two events that are crucial for successful telomere length maintenance. Thus, TPP1 is a very promising target for future studies focused on regulation of telomere length homeostasis at the molecular level. So far, shelterin and telomerase were thoroughly studied mainly on naked DNA in vitro. Quantitative biology of telomeres and associated nucleocomplexes could reveal new findings if we include nucleosomes into next investigations. Future quantitative studies of telomerase and shelterin associations and recruitment will contribute to further understanding of molecular origins of disease associated mechanisms and might be applied in the development of new molecular therapies. The preparation of a stable TEN domain of human telomerase in vitro seems to be a very challenging task that slows down further investigation leading to rational design of the inhibitor of telomerase recruitment via shelterin. The direct inhibition of telomerase catalytic activity is possible by GRN163L (Imetelstat) that is promising regarding the future development of combined biochemical approaches to eradicate tumor cells with less side effects.
